# Millennial-scale northern Hemisphere Atlantic-Pacific climate teleconnections in the earliest Middle Pleistocene

**DOI:** 10.1038/s41598-017-10552-2

**Published:** 2017-08-30

**Authors:** Masayuki Hyodo, Balázs Bradák, Makoto Okada, Shigehiro Katoh, Ikuko Kitaba, David L. Dettman, Hiroki Hayashi, Koyo Kumazawa, Kotaro Hirose, Osamu Kazaoka, Kizuku Shikoku, Akihisa Kitamura

**Affiliations:** 1 Research Center for Inland Seas, Kobe University, Kobe, 657-8501 Japan; 2 Department of Earth Sciences, Ibaraki University, Mito, 310-8512 Japan; 3 Hyogo Museum of Nature and Human Activities, Sanda, 669-1546 Japan; 4 Research Centre for Palaeoclimatology, Ritsumeikan University, Kusatsu, 525-8577 Japan; 5 Department of Geosciences, University of Arizona, Tucson, AZ 85721 USA; 6 Interdisciplinary Faculty of Science and Engineering, Shimane University, Matsue, 690-8504 Japan; 7 Department of Planetology, Kobe University, Kobe, 657-8501 Japan; 8 Research Institute of Environmental Geology, Chiba, 261-0005 Japan; 9 Institute of Geosciences, Shizuoka University, Shizuoka, 422-8529 Japan

## Abstract

Suborbital-scale climate variations, possibly caused by solar activity, are observed in the Holocene and last-glacial climates. Recently published bicentennial-resolution paleoceanic environmental records reveal millennial-scale high-amplitude oscillations postdating the last geomagnetic reversal in the Marine Isotope Stage (MIS) 19 interglacial. These oscillations, together with decoupling of post-reversal warming from maximum sea-level highstand in mid-latitudes, are key features for understanding the climate system of MIS 19 and the following Middle Pleistocene. It is unclear whether the oscillations are synchronous, or have the same driver as Holocene cycles. Here we present a high resolution record of western North Pacific submarine anoxia and sea surface bioproductivity from the Chiba Section, central Japan. The record reveals many oxic events in MIS 19, coincident with cold intervals, or with combined cold and sea-level fall events. This allows detailed correlations with paleoceanic records from the mid-latitude North Atlantic and Osaka Bay, southwest Japan. We find that the millennial-scale oscillations are synchronous between East and West hemispheres. In addition, during the two warmest intervals, bioproductivity follows the same pattern of change modulated by bicentennial cycles that are possibly related to solar activity.

## Introduction

Millennial-to-centennial scale rapid climate changes found in the North Atlantic sediments and Greenland ice cores^1–3^ greatly contribute to our understanding of the global climate system^4–6^. Some of these changes may be caused by ice sheet melting modulated by solar activity^2, 5^. Because centennial-to-decadal resolution records exist for Holocene and last-glacial climates, these have been the primary target for millennial scale variability studies. MIS 19 also attracts attention as an orbital analogue to the Holocene (MIS 1)^7, 8^, and as the interglacial that includes both the Early-Middle Pleistocene boundary^9, 10^ and a geomagnetic reversal. However, millennial-to-centennial scale rapid climate oscillations have rarely been discussed for MIS 19 due to the lack of high-resolution records^11–13^. The Kazusa Group in Chiba Prefecture, Japan, has potential to yield decadal-resolution climate records because of its high accumulation rates (a.r.). The Pleistocene fore-arc basin fill of the Kazusa Group was deposited in the western North Pacific (Supplementary Fig. S1) with a thickness of ca. 3,000 m and a mean a.r. of 2–3 m/ka^14, 15^. The Kokumoto Formation in the upper part of the Kazusa Group consists of continental shelf edge to continental slope deposits correlated with MIS 21 to 18^14–16^. The Chiba Section of the Kokumoto Formation has been studied extensively^9, 15, 17^, because it is a candidate for formal recognition as the Global Boundary Stratotype Section and Point (GSSP) for the base of the Middle Pleistocene stage. The TB2 core, drilled in the Chiba Section^18^, provides a continuous and high-resolution record of paleoenvironmental changes during MIS 19.

The sea-floor environment of this region of the Japan Sea, especially the degree of anoxia, is controlled by the strength of the East Asian monsoon. Under stronger monsoon conditions, the East China Sea’s low-salinity and nutrient-rich coastal water mass enters the Japan Sea through the Tsushima Strait^19^. High-resolution records of organic matter content and sediment color variation since the last glacial in a core recovered from the Japan Sea show millennial scale variation synchronous with Greenland ice core oxygen isotope data^19^, likely related to Dansgaard–Oeschger (D-O) cycles^3^. These data, together with terrestrial sediment records^20–23^, suggest the possibility of millennial-scale East-West hemispheric climatic teleconnections in the late Quaternary. The TB2 core sediments, deposited under anoxic conditions, provide a high-resolution record of magnetic susceptibility and Ca content for MIS 19. Both records show synchronized 10-m scale sinusoidal variations^18^ probably associated with orbital scale sea-level changes, and they contain many high-frequency oscillations possibly reflecting suborbital scale climate changes. Thus, the core is a good candidate for the investigation of millennial-to-centennial scale rapid climate oscillations during MIS 19 and their global extent.

## Results

### Magnetic data and the identification of oxic events

**Figure 1:**
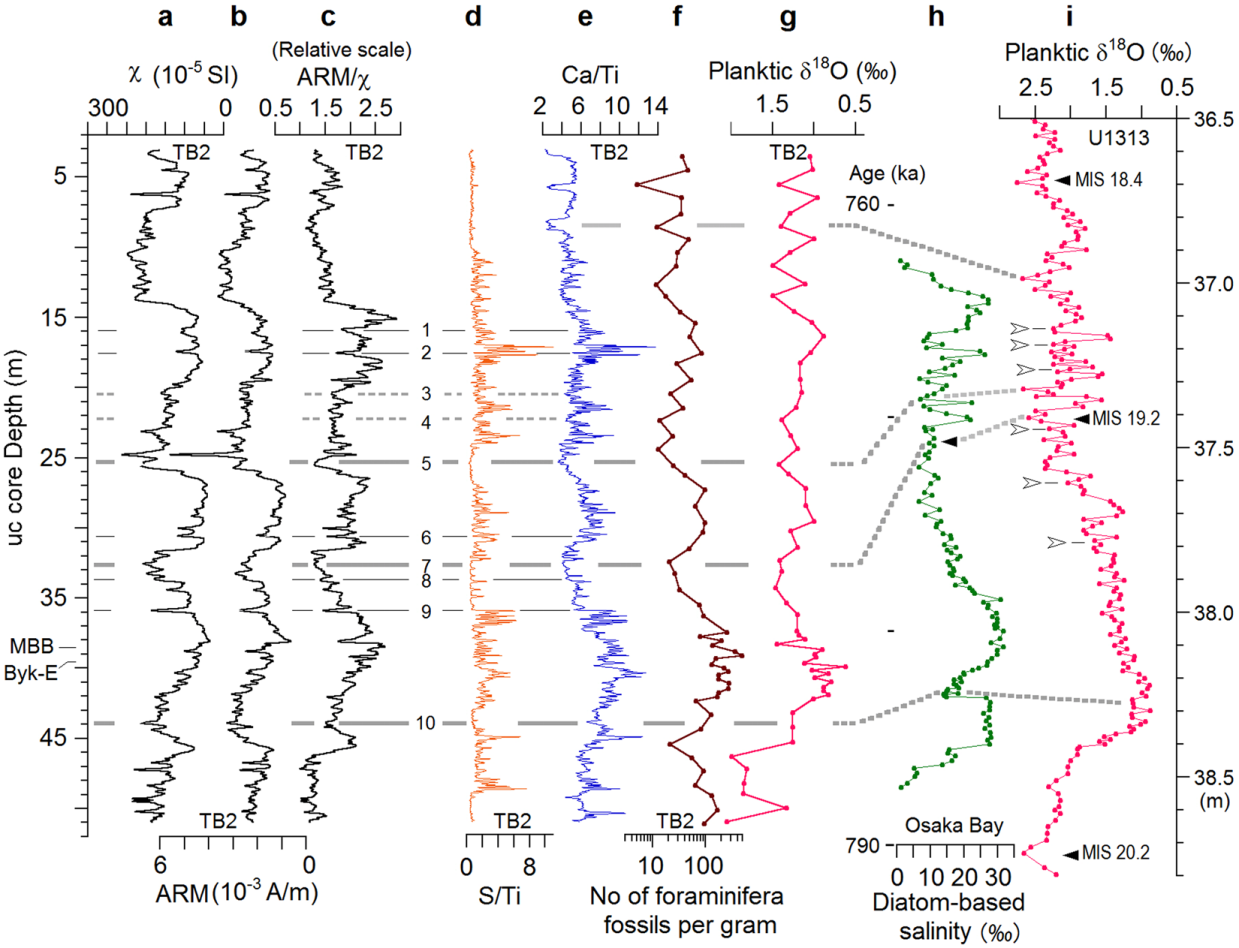
Magnetic, chemical and micropalentological data from core TB2, compared with paleoceanic data from other regions. The data from TB2 are (**a**) magnetic susceptibility (χ), (**b**) anhysteretic remanent magnetization (ARM), (**c**) ARM/ χ, a magnetic grain size proxy, (d) S/Ti ratio, a pyrite content proxy, (**e**) Ca/Ti ratio, (**f**) the number of foraminifera fossils per gram, and (**g**) planktic δ^18^O. The data from other regions are (**h**) diatom-based salinity from Osaka Bay^13^, and (i) planktic δ^18^O from U1313 in the mid-latitude North Atlantic^12^. The vertical axis for (**a**)–(**g**) is the u-channel core depth in m. The data of (**a**)–(**e**) are of 1-cm intervals. For (**d**) and (**e**), 7 point moving average data are plotted. The oxic events (or reduction in anoxia – see text for details), observed as broad or sharp minima in all of ARM/χ, S/Ti and Ca/Ti ratios, are numbered. The major oxic events that coincide with low sea-level events are tied by thick solid lines numbered 5, 7, and 10. Brief oxic events, less than 40 cm in thickness, are tied by thin solid lines numbered 1, 2, 6, 8, and 9. Weaker oxic events observed as broad minima are tied by thin broken lines numbered 3 and 4. The solid triangles in (**h**) and (**i**) show orbital scale sea-level lowstands. The open arrowheads in (**i**) show sporadic occurrences of iceberg discharge during the MIS 19 interglacial^12^. MBB: Matuyama-Brunhes boundary, Byk-E: Byakubi E tephra.

The TB2 core from the Chiba Section consists entirely of siltstones with many thin volcanic layers (Supplementary Fig. S2). Presence of a ferrimagnetic iron sulphide, greigite (Fe_3_S_4_), throughout the core suggests a persistent anoxic depositional environment^18^. Greigite is a precursor mineral of the paramagnetic mineral pyrite (FeS_2_), and both form in anoxic, sulphate-reducing sedimentary environments^24, 25^. Core TB2 includes both magnetite and greigite^18^, and lower magnetic susceptibility (χ) values indicate greater amounts of greigite and pyrite in core sediments (Supplementary Fig. S3). Lower χ values for iron sulphide-rich sediments are caused by dissolution of fine magnetite grains under anoxic conditions. The core’s χ values show quasi-periodic variations with narrow maxima and broad minima (Fig. 1a). Maxima (less anoxic or oxic events) occur at about 44 m, 33-32 m, and 26-25 m, alternating with minima (anoxic). Coherent variation between anhysteretic remanent magnetization (ARM) and χ show the response to changes in concentration of ferrimagnetic grains (Fig. 1b). The inverse correlation of ARM/χ, a magnetic grain size proxy, with χ and ARM (Fig. 1c) shows that anoxic intervals have small grain sizes probably due to the contribution of fine authigenic greigite particles, whereas the oxic intervals have reduced fine-grain growth, and thereby show coarser grain size signals than anoxic zones. The χ, ARM and ARM/χ values synchronously exhibit several sharp (30–40 cm thick) oxic events evidenced by grain coarsening. Volcanic layers at 24.8 and 6.3 m show maximum χ and ARM, but have no coarsening, probably affected by fine magnetite grains of volcanic origin.

### Coherent variation in magnetic, chemical, and micropaleontological data

Magnetic susceptibility and other magnetic parameters, all reflecting changes in the degree of anoxia (Supplementary Information), show 10-m scale sinusoidal variations synchronized with Ca content (Ca/Ti ratio), and reveal ten oxic events during MIS 19 (Fig. 1). Although core TB2 sediments contain low levels of allochthonous calcite, biogenic calcium carbonate is abundant, mostly coccoliths with rare foraminifera (Supplementary text, Fig. S5). Biogenic calcium carbonates therefore contribute significantly to calcium content. Quasi-periodic variation in Ca/Ti is well correlated with foraminifera fossil content, and with planktic δ^18^O values (Fig. 1e–g). We therefore infer that the Ca/Ti ratio primarily reflects biogenic calcium carbonate production, most likely representing primary production governed by nutrient levels in the local aquatic environment and by temperature changes^26^, both tied to orbital scale sea-level variations. The Ca/Ti ratio also exhibits suborbital-scale high-frequency changes which are synchronous with variation in the S/Ti ratio, an indicator of pyrite content (Fig. 1d). Both Ca content and S content drop to minimum values during oxic events. Thus, core TB2 provides records of orbital to suborbital scale paleoceanic environmental changes, coherent among different parameters.

### Synchronized millennial- and orbital-scale paleoceanic variation

**Figure 2:**
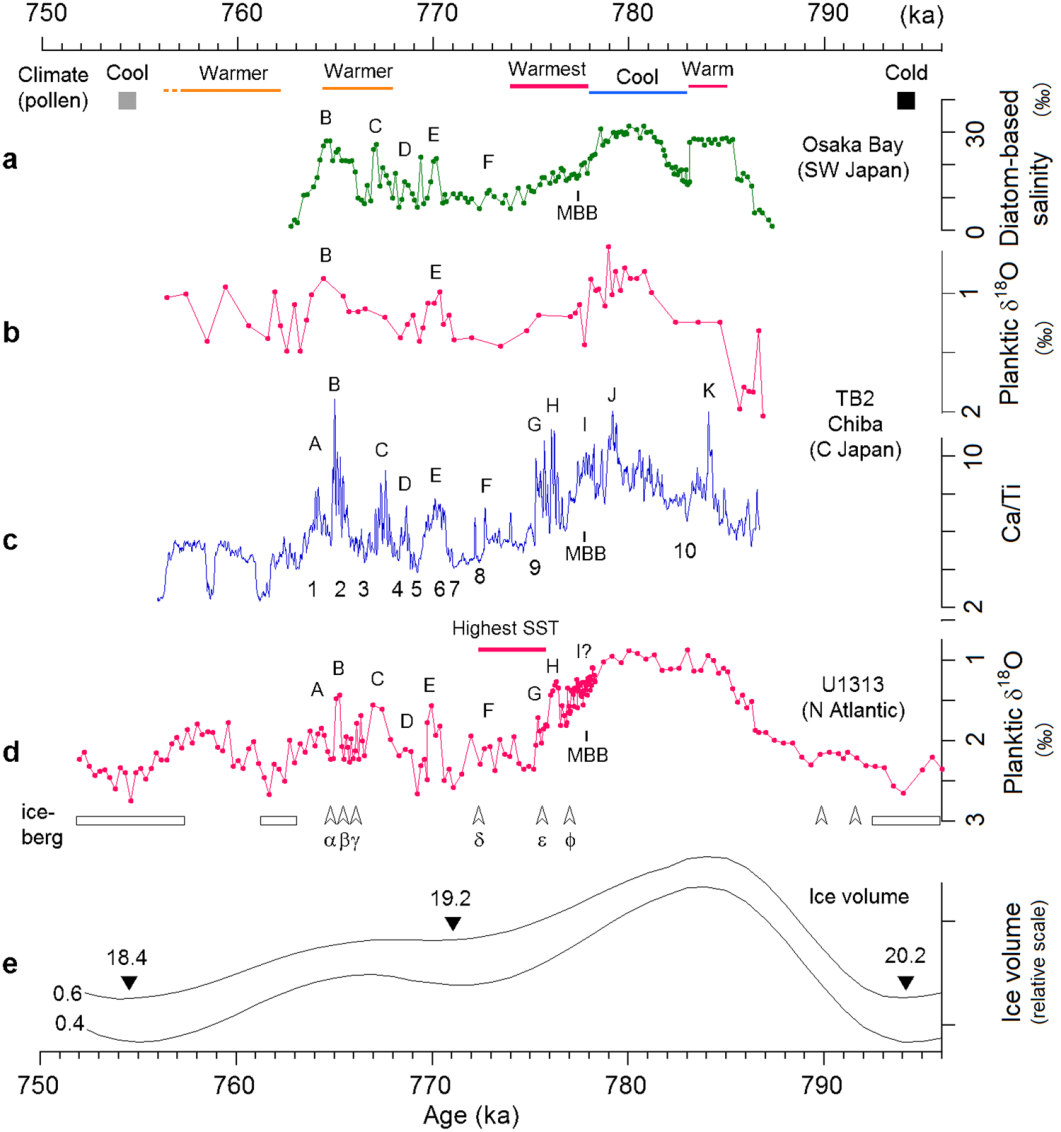
Age plots of paleoceanic environment proxies. (**a**) Diatom-based salinity from Osaka Bay^13^, (**b**) planktic δ^18^O from the Chiba Section (TB2), (**c**) 10-yr resolution Ca/Ti ratio from the Chiba Section (TB2) (see Methods), (**d**) planktic δ^18^O from IODP site U1313, mid-latitude North Atlantic^12^, and (**e**) ice volume variation, calculated at 15 ka for the mean time constant, and 0.6 and 0.4 for the nonlinearity parameter^46^ (Supplementary Information). In (**a**–**d**), the common millennial-scale features possibly reflecting sea-level rise and/or warming are labeled A to K. In (**a**), the climate features for Osaka Bay, shown by bars and solid squares, are based on pollen data^11^. In (**c**), the oxic events numbered 1 to 10 in Fig. 1 are shown. In (**d**), sporadic and continuous occurrences of iceberg discharge are shown by open arrowheads and open rectangles, respectively (after ref. 12). Short-lived sporadic iceberg discharge events during the MIS19 interglacial in Fig. 1 are labeled α to φ. For the plot of U1313 data, the MBB is shown at an age/depth corrected upward by 16 cm, an average lock-in depth for deep-sea cores^47^, but the MBB level is not corrected in the plots of Osaka Bay and Chiba (TB2) data with a.r. higher than 60 cm/ka.

**Figure 3:**
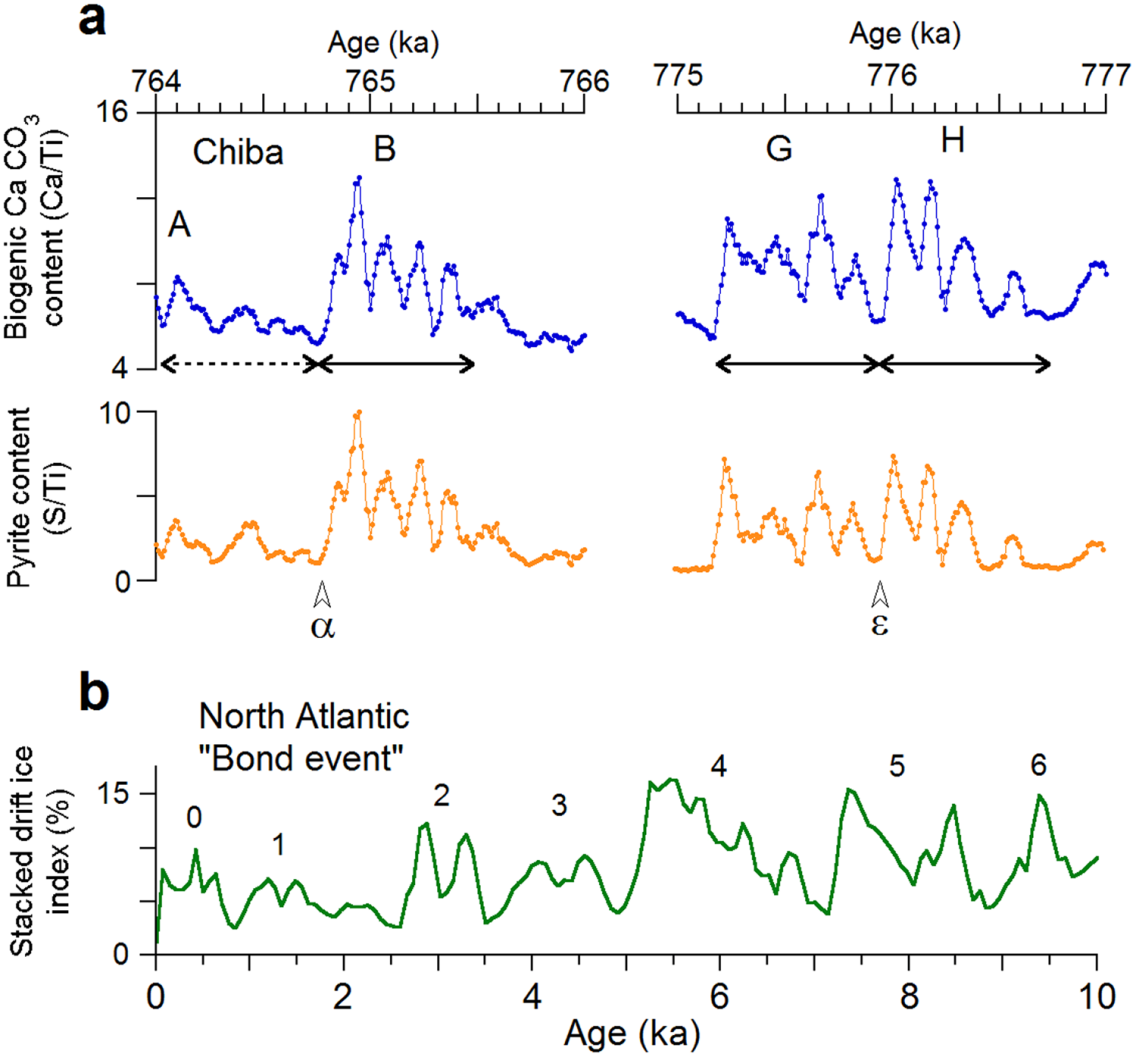
Comparison of similar climate variation patterns observed in the warmer intervals during MIS 19 and the Holocene. (**a**) The biogenic CaCO_3_ (Ca/Ti) and pyrite (S/Ti) contents in the intervals from 764 to 766 ka, and from 775 to 777 ka from the Chiba Section (TB2). The iceberg episodes (α, ε) observed at IODP site U1313 in the mid-latitude North Atlantic^12^ are transferred to the TB2 data by the correlation in Fig. 2. The double-headed arrows show events consisting of four bicentennial cycles. (**b**) Stacked drift ice index from the North Atlantic^2^.

The 10-yr resolution data of Ca content in core TB2 reveals millennial- and orbital-scale variation that is quite consistent with the sea-level proxy from Osaka Bay and planktic δ^18^O values from the mid-latitude North Atlantic (IODP Site U1313) (Fig. 2). The timing of the Matuyama-Brunhes magnetic polarity boundary (MBB) agrees well among the three records, and millennial-scale high-amplitude variation in the interval postdating the MBB is synchronous and similar in shape (peaks labeled A to H in Fig. 2). The high-resolution Ca and S content data reveal that bioproductivity and pyrite formation follow the same pattern of change spanning a period of about 800 yr: a gradual increase modulated by bicentennial cycles and terminated by a rapid decrease (e.g., events B, G, and H in Fig. 3). Events G and H may be related to the warmest climate in Osaka Bay and the highest SST in the mid-latitude North Atlantic (U1313), both just postdating the MBB. Event B coincides with the second warmest interval correlated to the MIS 19.1 highstand.

## Discussion

Ten oxic events during MIS 19 are observed in the core’s ARM/χ, S/Ti and Ca/Ti ratios, numbered 1 to 10 in descending stratigraphic order (Fig. 1). These oxic intervals are synchronous with cold events, some of which coincide with low sea-level events. Above 9 m core depth there are three large drops in the Ca/Ti ratio, but these have no comparable signals in either the S/Ti or ARM/χ, and thus do not reflect the occurrence of a strongly oxic environment. Biogenic calcium carbonate production (Ca/Ti ratio) and S or pyrite content (S/Ti ratio) fall to minimum values during oxic events, consistent with the magnetic data (Fig. 1). Because organic matter content is the major control on pyrite formation in normal (non-euxinic) marine sediments^25^, proxies of anoxia can be expected to correlate with bioproductivity-related parameters. Terrestrial nutrients derived from summer monsoon rainfall in the Kanto region of central Japan strongly affect primary productivity at the core site (Supplementary Fig. S1). Studies have shown that the East Asian summer monsoon was weaker than average in glacial periods and during the stadials of the D-O cycle^19–23^. Therefore oxic events at the TB2 core site may have been affected by monsoon variability on both millennial-centennial scales as well as on an orbital scale.

The orbitally tuned high-resolution diatom record obtained from a core sequence with a mean a.r. of 63 cm/ka in Osaka Bay, Southwest Japan^13^, provides several coherent sea-level proxies, among which the salinity estimated from diatom assemblage data is most comprehensive (Fig. 1h). Another high-resolution record, planktic δ^18^O obtained at 1-cm depth resolution from IODP site U1313, reveals detailed paleoceanographic variations in the mid-latitude North Atlantic^12^ (Fig. 1i). Both records show multiple high-amplitude changes just after lowstand MIS 19.2. The major oxic events 5, 7 and 10 in core TB2 are well correlated with the sea-level lowstands/cold events shown by salinity minima in Osaka Bay and δ^18^O maxima at U1313. Oxic event 10, at about 44 m, is correlated with the brief sea-level fall event clearly seen in the Osaka Bay record. In addition, the high-amplitude changes postdating lowstand MIS 19.2 seem to be correlated with the Ca/Ti ratio (Fig. 1). The TB2 and U1313 paleoceanic records are dated based on tuning to the Osaka Bay record from an interval of fine clay deposition with uniform accumulation rates (Supplementary text, Fig. S8). The temporal variation in sea-level, bioproductivity, and paleoclimate proxies from the three sites are consistent with each other, and with global ice volume records (Fig. 2). They even show coincident millennial-scale changes. The MBB is present in all records during the decrease in sea-level toward lowstand MIS 19.2.

The covariation of Ca/Ti ratio with sea-level proxies in Fig. 2 suggests that the nutrient flux and temperature controlling biogenic calcium carbonate production are indirectly related to sea-level. Thus, this ratio may be regarded as a sea-level proxy. The curves in Fig. 2a–d show short-term (500–2000 yr) features labeled A to K, superimposed on orbital modulation. Events B, C, D, E and F observed in Osaka Bay primarily reflect sea-level variation associated with warming. Event B is correlated with highstand MIS 19.1. Events A, G, H and I, seen in the North Atlantic and Chiba but not clear in Osaka Bay, probably reflect warming without significant sea-level changes. Events J and K, observed only in Chiba, cannot be confirmed to be global at present. Absence of C and F in the TB2 δ^18^O record may be due to the low-resolution of the data. The benthic diatom sea-level proxies in Osaka Bay^13^ show event F, but C is part of a broad increase. C may be a broad rise like that in the TB2 δ^18^O record. Therefore, the clear signals of C in the TB2 Ca/Ti and U1313 δ^18^O data seem to primarily reflect warming climate.

Oxic events 1 to 10 can be correlated with cooling events, some of which are associated with low sea-level events. In contrast, events A to K seem to correlate with warming and high sea-level intervals. The core proxies respond differently to climate variation; the magnetic proxy is most sensitive to cooling/low sea-level events, while the elemental ratios respond more to warming/high sea-level events. Some of these features may be related to the iceberg discharge episodes observed at U1313^12^ (Fig. 2d). During MIS 19, the first and strongest episode (ϕ), occurred just after the MBB, followed by sporadic occurrences of five small-sized episodes (α to ε). The slight increase in δ^18^O value at every episode seems to reflect decreases in water temperature. The difference in δ^18^O value between highstand MIS 19.3 at about 780 ka and lowstand MIS 19.2 at about 771 ka is about 1.5‰, equivalent to 75 m in sea-level estimated assuming a constant isotopic rate of change^27^, which is much larger than that of TB2 (about 0.5‰, equivalent to 25 m in sea-level). The large difference can probably be ascribed to cold melt water at U1313 after episode (ϕ).

Sea-level in Osaka Bay during MIS 19 ranges from −48 ± 4 m to about −20 m in elevation^13^. The sea-level for the MIS 19.2 lowstand is within this range, consistent with the sea-level difference between highstand MIS 19.3 and lowstand MIS 19.2 estimated from core TB2. During MIS 19, the sea-levels associated with events B to F can be placed in descending order based on the core data: MIS 19.3 highstand, B (MIS 19.1 highstand), E, (C), D, F, and the MIS 19.2 lowstand. Although the precise amplitudes of the millennial scale sea-level variations are unknown, these variations may have been caused by ice sheet changes. For example, a simulation of Heinrich events^28^, related to large-scale surges of the Laurentide ice sheet^29^, suggests that a total ice volume discharge corresponding to 5–10 m of sea-level rise may have occurred within several hundred years in each event^30^. Similar amplitude sea-level changes may have occurred during MIS 19. Event A may be a split from B, possibly caused by iceberg discharge episode α. During the transition toward the MIS 18.4 lowstand, a sea-level rise/warming occurred, centered at 757–759 ka (Fig. 2). Its maximum sea-level should be lower than −48 ± 4 m, due to absence of corresponding seawater incursion into Osaka Bay^13^.

Wavelet analysis of the biogenic calcium carbonate content (Ca/Ti) reveals that centennial-scale periodicities are clearly dominant during two intervals, about 764.5–766 ka and 775–777 ka. The spectral power is especially concentrated at period bands from about 100–300 yr and 600–1000 yr in these intervals – corresponding to events B, and G–H (Supplementary Fig. S9). Events B, G and H are closely related to intervals of warm climates. Events G and H occurred during the warmest interval, when *Quercus* (*Cyclobalanopsis*), evergreen oak, dominates the Osaka Bay pollen record^11^, and the alkenone-based SST reached a maximum in the mid-latitude North Atlantic^12^ (Fig. 2). This interval corresponds to the post-magnetic-reversal warming also observed in Italy, Israel and Lake Baikal^31, 32^. This unusual delay in temperature maximum until well after highstand MIS 19.3 was probably due to an increase in cloud-cover induced by cosmic rays^33–35^ during the weak geomagnetic field interval^11, 36^. Event B occurred during the second warmest phase correlated to highstand MIS 19.1^11, 32^. These events have the same variation pattern as that of biogenic production/temperature; temperatures gradually increase through four nearly bicentennial oscillations, terminated by sharp decreases that span only 150 yr, 60 yr, and 60 yr for B, G, and H, respectively (Fig. 3). The duration of these cycles and ages of the transitions depend on the mean a.r. between control points in the age model, 114 cm/ka for B and 85 cm/ka for G and H. Nevertheless, the total duration of all four cyclical events is 750–800 yr, with an average periodicity of 190–200 yr.

The terminations of B and H seem to have coincided with iceberg discharge episodes α and ε at U1313. Pyrite (S) content covaries strongly with Ca content (Fig. 3), suggesting quick (<10 yr) submarine anoxic environmental response to the sea surface biogenic production. Events A and B both show a pattern of gradual increase and sudden decrease. They span about 750 yr, and may be a pair like G and H. These periodicities are consistent with the wavelet power spectra (Supplementary Fig. S9). Bicentennial cyclicity is also observed around warm events C and K. This bicentennial periodicity may be ascribed to the deVries solar cycle^37, 38^. Periodicity in sea surface biogenic production (Ca/Ti) could respond to solar irradiation directly, or indirectly through climate. There may have been a solar cycle forcing amplification mechanism^39^ that only worked during warm periods in MIS 19. The successive events G and H span 1550 yr in total, which is close to the Bond cycle of 1470 ± 500 yr that is thought to be of solar origin^1, 2, 40–42^. Bond events consist of a gradual increase and sudden decrease in ice rafted debris (IRD) that are interpreted as gradual cooling terminated by sudden warming (Fig. 3b), whereas events B, G, and H show gradual warming terminated with sudden cooling. Unlike the major iceberg episodes in MIS 20 and 18 glacial periods when ice sheets expanded, episodes α to ϕ during MIS 19 are in warm intervals (Fig. 2). This suggests that IRD episodes can be induced by warming and attributed to self-sustained ice sheet oscillations^43^.

The millennial-scale sea-level and/or climate oscillations postdating the MBB include those affected by the geomagnetic field and/or modulated by solar cycles, and are synchronous between the mid-latitude North Atlantic and western North Pacific. These features can contribute to the high-resolution climatostratigraphy as well as elucidation of the climate system in the earliest Middle Pleistocene. Here we note that the high-resolution benthic δ^18^O record at U1313 lacks millennial scale oscillations^12^. There may be a high-cut filtering mechanism in the benthic δ^18^O changes in the North Atlantic, perhaps related to melt water dynamics. However, such a mechanism does not seem to apply across the entire North Atlantic, because some low-resolution records^44, 45^ show a small number of fluctuations in benthic δ^18^O values just postdating the MBB.

## Methods

### Sample preparation

In the previous study^18^, u-channel samples of 2 × 2 × 100 cm^3^ were prepared from core TB2 ranging in depth from 3 to 51 m, excluding the topmost 3-m weathered mud/tephra layers, lowermost 2-m sandstone layers, and thin (3 to 36 cm) turbidite sand layers. Discrete cubic specimens, 2 cm on a side, were also collected for magnetic analyses. Block samples of about 200 cm^3^ were collected at 25-cm to 1-m depth intervals from half core sections to collect foraminifera fossils for oxygen isotope analyses. To support oxygen isotope data from the core, we took block samples directly from the Chiba Section (Tabuchi) exposed along the Yoro River (Supplementary Figs S1, S2), and collected rare benthic foraminifera fossils from the core.

### Experiments

The main data source of this study is the 1-cm resolution magnetic and chemical data reported in Hyodo *et al*.^18^. To enlarge the magnetic data set and our understanding of environmental magnetism, we added magnetic experiments using the u-channel and discrete samples (Supplementary Fig. S3). The experiments include measurements of frequency dependence of magnetic susceptibility (χ_FD_) and anhysteretic remanent magnetization (ARM). We then conducted SEM observations of sediments to check for the presence of framboidal pyrite, using a TM3000 Miniscope of Hitachi High-Technologies Co. In addition, stable isotope analyses were conducted on foraminefera fossils collected from core TB2 and block samples from the Chiba Section (Supplementary Figs S1, S2).

The frequency dependence of magnetic susceptibility (χ_FD_) was determined on discrete specimens using a SM-100 ZH-Instrument susceptibility meter at low frequency (0.5 k Hz) (χ_LF_) and high frequency (17 k Hz) (χ_HF_) magnetic susceptibility. χ_FD_ is calculated as: χ_FD_ = (χ_LF_ − χ_HF_)/ χ _LF_ × 100. Using u-channel samples, anhysteretic remanent magnetization (ARM) is imparted every 1-cm depth interval with a peak alternating demagnetization field of 80 mT superimposed on a DC-biased field of 50 mT, and measured using a 2 G pass-through cryogenic magnetometer with a sensor-response function of about 4 cm width. The data for 5-cm depth intervals at both ends of each u-channel sample were not used in consideration of the sensor width. Isothermal remanent magnetization (IRM) acquisition experiments were conducted with pieces of sediment using a VSM of MicroMag 3900.

For oxygen isotope (δ^18^O) analyses, we picked planktic foraminifera *Globorotalia inflata*, the species most abundant throughout the core (Supplementary text, Fig. S4). Using twenty tests per horizon on average, stable oxygen isotope ratios were measured using an automated carbonate preparation device (KIEL-III) coupled to a gas-ratio mass spectrometer (Finnigan MAT 252). The isotope ratio measurement is calibrated based on repeated measurements of NBS-19 and NBS-18 and precision is ±0.10‰ for δ^18^O (1 sigma). Reported values are in per mil (‰) relative to VPDB. During the fossil collection process, we calculated the number of foraminifera fossils per 1 gram of dry weight. Due to low content rates of benthic foraminifera fossils and limited available volume of the sediments, benthic oxygen isotope data are unavailable from the core. To support the core data, we used block samples from the outcrop of the Chiba Section. We collected benthic foraminifera *Bolivinita quadrilatera* fossils, and measured δ^18^O with 14 fossils per horizon on average.

The 1-cm resolution magnetic susceptibility data was obtained by core-logging using split 1-m core sections^18^ (Supplementary Fig. S2). For comparison with data from u-channel samples, the magnetic susceptibility data for the intervals of turbidite sand layers were removed and the depth scale of the core is adjusted to the u-channel one.

We use the 1-cm depth interval data of contents for Ti, Ca, and S obtained from the u-channel sample using the X-ray Fluorescence core logger (JSX-3600 CAZ (TATSCAN-F2), with a beam radius of 0.7 mm) at Center for Advanced Marine Core Research, Kochi University^18^. Ti in marine sediments is thought to be of terrigenous origin, and therefore S/Ti and Ca/Ti ratios are used as parameters of S and Ca contents (Supplementary Fig. S5e). Polarizing microscope observations of bulk sediments with smear slides reveal that most calcitic particles are coccoliths, and rarely foraminifera (Supplementary Fig. S5a–d). Most of detrital grains are silicates. We could not find any detrital carbonates, which is consistent with the fact that limestone basement is rarely exposed in the hinterland of the core site (Supplementary Fig. S1b).

### Age model

Age models are constructed for the TB2 and U1313 data by tuning to the astronomically dated sea-level proxy record from Osaka Bay. The record is suitable as a reference because this interval is dated with six age control points, four of which are within MIS 19 including lowstand MIS 19.2^13^. Many δ^18^O records of deep-sea cores are lacking in both a MIS 19.2 signal and a MIS 19 termination^32^, leading to cases in which the beginning of MIS 19 is the only age control for the MIS 19 interglacial, with the next control point not until the MIS 18.4 lowstand. Another reason to use the Osaka Bay core is that the marine clay layers have a very uniform a.r. In the Osaka Basin, many core studies demonstrate that Pleistocene sediments have been deposited at uniform rates at least since MIS 37^13, 48^. Uniform deposition is probably related to uniform displacement of basement rocks across active faults^13, 49^. Linear age models have therefore been proposed for interglacial marine layers, and their validity has been demonstrated by the agreement with marine isotope stacks or ice volume models^32, 48^. For MIS 19, the astronomical age model^13^ agrees very well with the linear age model^11^ with differences less than 1.2 kyr (0.4–0.5 kyr on average).

The correlations of oxic events to low sea-level and/or cooling events are the primary tie-lines in Supplementary Fig. S6, and the low sea-level events 9a, 9b, 10a, and 10b are added as common features. Furthermore, the most negative δ^18^O value in the U1313 and TB2 cores (event P) is correlated to the highest sea-level highstand in Osaka Bay, an interval identified with the highest salinity, maximum pelagic diatoms, and minimum marine benthic diatoms^13^. Event P for TB2 is confirmed by the benthic δ^18^O data from hand samples collected from the Chiba Section (Supplementary Fig. S7).

As in Supplementary Fig. S6, events 1, 2, 3, 4, 5, 7, 8, 9 and 10, and prominent features 9a, 9b, P, 10a and 10b are common to U1313, Osaka Bay or TB2. The ages of tie-lines within MIS 19 are used as age controls for TB2 and U1313. Besides these age control points, we add sea-level lowstands MIS 20.2 and 18.4 as control points for U1313. These lowstands are adjusted to the ice volume maxima calculated with the same conditions as those for the global isotope stack LR04^50^ and the Osaka Bay data^13^. The tie-lines ii and i (Supplementary Fig. S6) are dated by linear interpolation between lowstand 18.4 and tie-line 1. The age controls are plotted against depth in Supplementary Fig. S8. Mean a.r. for TB2 and U1313 are 156 cm/ka and 5.16 cm/ka, respectively. The high a.r. at about 8–16, 25–33, and below 44 m in core TB2 should be ascribed to low sea-levels (Supplementary text). The U1313 record has no such high a.r. related to low sea-levels, but a high a.r. at about 37.5–38.2 m, which is similar to the increase in a.r. around highstand MIS 19.3 in the high-latitude North Atlantic, possibly related to the mid-latitude cooling event^32^ (Supplementary Fig. S10).

An error of ±0.5 kyr estimated for age controls of the Osaka Bay data^13^ is also applied to the age models for both of TB2 and U1313. In addition, the correlation depths of features have uncertainty depending on the data interval. Thus, the U1313 data with relatively large time intervals have an additional error of ±0.5 ky, and consequently have a total error of ±1 kyr.

### Depth to age conversion

Depths of TB2 and U1313 are converted to ages by linear interpolations between age controls and extrapolations beyond ages at both ends using the adjacent a.r. values. The data intervals of the u-channel samples of TB2 are 7.6 yr on average, ranging from 12.5 to 2.5 yr. For the Ca/Ti ratio, a biogenic calcium carbonate production proxy, decadal data were smoothed with a 70-yr moving average, resulting in a reduction of the number of data from 4,807 to 3,080 points. The same calculation was made for the S/Ti ratio, a pyrite content proxy, used in Fig. 3.

The age models date the Matuyama-Brunhes polarity boundary (MBB) at 777–778 ka for the TB2 and 777–779 ka for the U1313 data, consistent with that from Osaka Bay^13^. These age estimates agree within errors with the mean Ar^40^/Ar^39^ date of 776 ± 2 ka from Matsuyama-Brunhes transitionally magnetized lavas^51^, and with an estimated MBB age of 770.2 ± 7.3 ka from the U-Pb dating of the Byk-E tephra of the Kokumoto Formation^52^.

## Electronic supplementary material

Supplementary Information
