# Supplementary material for: Millennial-scale northern Hemisphere Atlantic-Pacific climate teleconnections in the earliest Middle Pleistocene
Source: Sci Rep. 2017 Aug 30;7:10036. doi: 10.1038/s41598-017-10552-2 (PMC5577287; doi:10.1038/s41598-017-10552-2)
Supplement: Supplementary file 1 — Supplementary Information [file 41598_2017_10552_MOESM1_ESM.pdf]

Supplementary information

Millennial-scale northern Hemisphere Atlantic-Pacific climate teleconnections in the earliest Middle Pleistocene

Masayuki Hyodo[1] ,★Balázs Bradák[1], Makoto Okada[2], Shigehiro Katoh[3], Ikuko Kitaba[4], David L. Dettman[5], Hiroki Hayashi[6], Koyo Kumazawa[7], Kotaro Hirose[1], Osamu Kazaoka[8], Kizuku Shikoku[6], Akihisa Kitamura[9]

[1] Research Center for Inland Seas, Kobe University, Kobe 657-8501, Japan

[2] Department of Earth Sciences, Ibaraki University, Mito 310-8512, Japan

[3] Hyogo Museum of Nature and Human Activities, Sanda 669-1546, Japan

[4] Research Centre for Palaeoclimatology, Ritsumeikan University, Kusatsu 525-8577, Japan

[5] Department of Geosciences, University of Arizona, Tucson, AZ 85721, USA

[6] Interdisciplinary Faculty of Science and Engineering, Shimane University, Matsue 690-8504, Japan

[7] Department of Planetology, Kobe University, Kobe 657-8501, Japan

[8] Research Institute of Environmental Geology, Chiba 261-0005, Japan

[9] Institute of Geosciences, Shizuoka University, Shizuoka 422-8529, Japan

★e-mail: mhyodo@kobe-u.ac.jp

Geological setting

The Kazusa Group, Lower and Middle Pleistocene sedimentary successions, is exposed in the central part of the Boso Peninsula in Chiba Prefecture, central Japan (Fig. S1). It unconformably overlies the Miocene and Pliocene marine sediments of the Miura Group, and conformably underlies the Middle and Upper Pleistocene Shimousa Group comprising shallow marine to paralic sediments[1, 2]. The Kazusa Group is a fill of the Plio-Pleistocene forearc basin that developed in response to the west-northwestward subduction of the Pacific plate beneath the Eurasia plate at the Izu-Bonin trench[1]. The basin began to uplift at about 1 Ma[3]. The Kokumoto Formation in the group consists of continental shelf edge to continental slope deposits correlated with MIS 21 to 18[1, 2, 4]. Core TB2 was drilled about 200 m east of the Chiba (Tabuchi) Section along the Yoro River (Fig. S1), and it spans the middle part of the formation[5].

Limestone basement rocks are poorly exposed in the Kanto Region (Fig. S1). The total exposure area is only 0.05 % of that of the present Kanto Region, the probable maximum catchment area for the TB2 core site. If the paleogeography of the area during MIS 16-17 can be used as a guide (revealed by the distribution of the Kaisho-Kamitakara tephra)[5], the MIS 19 terrestrial basin was approximately 1/2 to 1/3 of the present basin, and limestone exposure is estimated to occupy only 0.1 to 0.15 % of the basin area. Therefore, calcareous clastic deposition in the Kazusa Group was most likely very minor.

Magnetic data

The previous study of core TB2 includes paleomagnetic analyses[6]. The thermal demagnetization experiments show that natural remanent magnetizations are unblocked at 200–300 and 590 ºC (Fig. S3). Isothermal remanent magnetization acquisition (IRM) experiments reveal that carriers are low coercivity magnetic minerals (Fig. S3). These results suggest that the component unblocked at 200–300 ºC is carried by greigite[7], and that at 590 ºC by magnetite. In addition, during thermal treatments magnetic susceptibility of samples greatly increases above 350 ºC, which suggests thermal conversion of iron sulphide minerals (pyrite and greigite) to magnetite.

Magnetic susceptibility (χ) and the frequency-dependence of magnetic susceptibility (χ_{FD}) show inverse correlation (Fig. S3). Namely, high χ sediments have fewer paramagnetic grains, pyrite. We estimated the contributions of magnetite and greigite to the saturation isothermal remanent magnetization (SIRM) by IRM component analysis using IRMUNMIX software[8]. As a result, it was shown that the greigite content roughly covaries with pyrite.

Based on the χ_{FD} result, we conducted SEM observations of framboidal pyrites for different χ value sediments, and easily confirmed that high χ sediments contain lower amounts of pyrite, and vice versa (Fig. S3).

Foraminifera fossils used for isotope analysis

For the stable isotope analysis, benthic foraminiferal species *Bolivinita quadrilatera* and planktic foraminiferal species *Globorotalia inflata* were picked from the 125–250 µm size fraction. To avoid artifacts from the ontogenetic effects of planktic foraminifera, we employed adult specimens of *G. inflata* distinguished by its encrusted smooth surface structure (Fig. S4, 3a–c and 4a–c). For selected samples, foraminiferal preservation was examined using a scanning electron microscope (SEM) (JCM-5000: JEOL Co. LtD., Japan). The SEM observation reveals benthic foraminifera fossils are well preserved with no infilling cements or secondary overgrowth (Fig. S4, 1a–b and 2a–b). Some specimens are partly covered by silty materials (Fig. S4, 1a–b), which are easily removed by the cleaning process for the isotope analysis. In the planktic foraminiferal specimens of *G. inflata,* many were more or less infilled by silty sediment (Fig. S4, 3a–c and 4a–c). This infilling material was completely removed by our ultrasonic cleaning process (Fig. S4, 4d–e).

Age model and accumulation rates

The age model (Fig. S8), based on the correlations in Fig. S6 (see methods), suggests that the intervals 785–771 ka, 768–766 ka, 765–764 ka, and after 762 ka have low accumulation rates (a.r.) of about 80 to 130 cm/ka. These intervals correspond to high sea-levels in Osaka Bay (Fig. S6) as inferred from diatom assemblage data[9]. The low a.r. are probably caused by the inland shift of the shore zone away from the TB2 core site due to transgression. On the other hand, the intervals before 785 ka and 771–768 ka, 776–765 ka, and 764–762 ka have high a.r: 200 to 400 cm/ka (Fig. S8). The a.r. are calculated based on the depth of u-channel samples, where all thick sand layers and tephra layers have been removed from the calculation. Therefore, the lithology used for a.r. calculation is based entirely on silts, except for the presence of thin tephra layers[6]. Note, however, that the lowermost interval of high a.r. (below 44 m in depth and before 785 ka in age) in the u-channel sample is a zone intercalating turbidite sand layers in the original core sample (Fig. S2). The high a.r. may be related to turbidity currents, which may have brought additional silt-sized clastics to the core site that were not removed when turbidites were excluded. The diatom data[9] show that the high a.r. intervals generally match those dominated by reworked fossils of the

distinctive diatom species *Actinocyclus ingens*[10] (Fig. S8b). The high a.r. and deposition of reworked diatom fossils may be related. Both could occur at times of low sea-level. The diatom data of core TB2 will be published elsewhere.

Ice volume model

Ice volume (Fig. S9a) is calculated using the model of Imbrie and Imbrie[11], using insolation at 65ºN for June 21[12]. 15 ka is used for the mean time constant (Tm), and 0.6 and 0.4 for the nonlinearity parameter (b). The values of 15 ka for Tm, and 0.6 for b are those adopted for the global benthic oxygen isotope stack LR04[13].

Wavelet analysis

Wavelet analysis was conducted on the time series data of Ca/Ti to examine periodicity of biogenic calcium carbonate production. To focus on centennial-scale periodicity, low frequency components < 1/1010 yr were filtered out before the analysis. The result reveals that there are two intervals, 764.5−766 ka and 775−777 ka, when centennial-scale periodicities are dominant (Fig. S9). Spectral power is concentrated at period bands of 100−300 yr, and 600−1000 yr in both intervals. These two intervals correspond to warm (and high sea-level) events B and G−H, respectively. In addition, similar centennial-scale periodicities are observed at about 767−768 ka (event C) and 783−785 ka (event K). These intervals also coincide with warm events.

Reference

1. Ito, M. & Katsura, Y. Inferred glacio-eustatic control for high-frequency depositional sequences of the Plio-Pleistocene Kazusa Group, a forearc basin fill in Boso Peninsula, Japan. *Sedimentary Geology* **80**, 67–75 (1992).
2. Kazaoka *et al.* Stratigraphy of the Kazusa Group, Boso Peninsula: An expanded and highly-resolved marine sedimentary record from the Lower and Middle Pleistocene of central Japan, *Quat. Int.* **383**, 116-135 (2015).
3. Kaizuka, K. Quaternary crustal movements in Kanto, Japan. *J. Geography* **96**, 223–240 (1987) (in Japanese with English abstract).
4. Pickering *et al*. Galcioeustatic control on deep-marine clastic fore arc sedimentation, Pliocene-mid-Pleistocene (c. 1180–600 ka) Kazusa Group, SE Japan. *J. Geol. Soc. London* **156**, 125–136 (1999).
5. Suzuki, T. Kaisho-Kamitakara tephra erupted from the Hida Mountains in the early half of the Middle Pleistocene and its significance for geomorphic chronology of Central Japan. *Geogr. Rev. of Japan* **73A**, 1-25 (2000) (in Japanese with English

abstract).
6. Hyodo *et al.* High resolution stratigraphy across the early–middle Pleistocene boundary from a core of the Kokumoto Formation at Tabuchi, Chiba Prefecture, Japan. *Quat. Int.* **397**, 16–26 (2016).
7. Robert, A.P. Magnetic properties of sedimentary greigite (Fe_3S_4). *Earth Planet. Sci. Lett.* **134**, 227-236 (1995).
8. Heslop, D., Dekkers, M.J., Kruiver, P.P. & Van Oorschot, I.H.M. Analysis of isothermal remanent magnetisation acquisition curves using the expectation-maximisation algorithm. *Geophys. J. Int.* **148**, 58–64 (2002).
9. Tanaka, I. Hyodo, M., Ueno, Y., Kitaba, I. & Sato, H. Detailed variations in diatom assemblages from a core of the Kokumoto Formation, the Kazusa Group from the Chiba section, central Japan. *Abstract of the 123th meeting of the Geological Society of Japan, Tokyo*, 287 (2016). [in Japanese]
10. Cherepanova, M.V., Pushkar, V.S., Razjigaeva, N., Kumai, H. & Koizumi I. Diatom biostratigraphy of the Kazusa Group, Boso Peninsula, Honshu, Japan. *The Quat. Res. (Daiyonki Kenkyu)* **41**, 1-10 (2002).
11. Imbrie, J. & Imbrie, J.Z.. Modeling the climate response to orbital variations. *Science* **207**, 943–953 (1980).
12. Laskar, J. *et al.* A long term numerical solution for the insolation quantities of the Earth. *Astron. Astrophys.* **428**, 261–285 (2004).
13. Lisiecki L.E. & Raymo, M.E. A Pliocene–Pleistocene stack of 57 globally distributed benthic $\delta^{18}O$ records. *Paleoceanography* **20**, PA1003 (2005).
14. Geological Survey of Japan, AIST (ed.) Seamless digital geological map of Japan 1: 200,000. May 29, 2015 version. *Geological Survey of Japan, National Institute of Advanced Industrial Science and Technology* (2015).
15. Head, M.J. & Gibbard, P.L. Early-middle Pleistocene transitions: linking terrestrial and marine realms. *Quat. Int.* **383**, 7–46 (2015).
16. Kazaoka, O. *et al.* Detailed Litho-Stratigraphy of Kokumoto Formation, Kazusa Group, Lower－Middle Pleistocene Boundary in the Chiba Section , central Japan: For proving stable sedimentation. *Abstract of the Japan Association for Quaternary Research 2016 Meeting,* 39 (2016)
17. Maegakiuchi *et al.* Brief sea-level fall event and centennial to millennial sea-level variations during Marine Isotope Stage 19 in Osaka Bay, Japan. *J. Quat. Sci.* **31**, 809–822 (2016).
18. Ferretti, P. *et al.* The Marine Isotope Stage 19 in the mid-latitude North Atlantic Ocean: astronomical signature and intra-interglacial variability. *Quat. Sci. Rev.* **108**,

95–110, (2015).

19. Suganuma, Y. *et al.* Age of Matuyama–Brunhes boundary constrained by U-Pb zircon dating of a widespread tephra. *Geology* **43**, 491-494 (2015).
20. Torrence, C. & Compo, G.P. A practical guide to wavelet analysis. *Bull. Amer. Meteor. Soc.* **79**, 61-78 (1998).
21. Shackleton, N. Timescale Calibration, ODP 677. IGBP PAGES/World Data Center-A for Paleoclimatology Data Contribution Series # 96-018. *NOAA/NGDC Paleoclimatology Program, Boulder CO, USA* (1996).
22. Bassinot, F.C., Labeyrie, L.D., Vincent, E., Quidelleur, X., Shackleton, N.J. & Lancelot, Y. The astronomical theory of climate and the age of the Brunhes–Matuyama magnetic reversal. *Earth Planet. Sci. Lett.* **126**, 91–108 (1994).
23. Channell, J.E.T. & Raymo, M.E. Paleomagnetic record at ODP Site 980 (Feni Drift, Rockall) for the past 1.2 Myrs. *Geochem. Geophys. Geosys.* **4**, 1033. doi:10.1029/2002GC000440 (2003).
24. Channell, J.E.T. & Kleiven, H.F. Geomagnetic palaeointensities and astrochronological ages for the Matuyama–Brunhes boundary and the boundaries of the Jaramillo Subchron: palaeomagnetic and oxygen isotope records from ODP Site 983. *Philosophical Transactions of The Royal Society of London Series A : Mathematical Physical and Engineering Sciences* **358**, 1027‑1047 (2000).

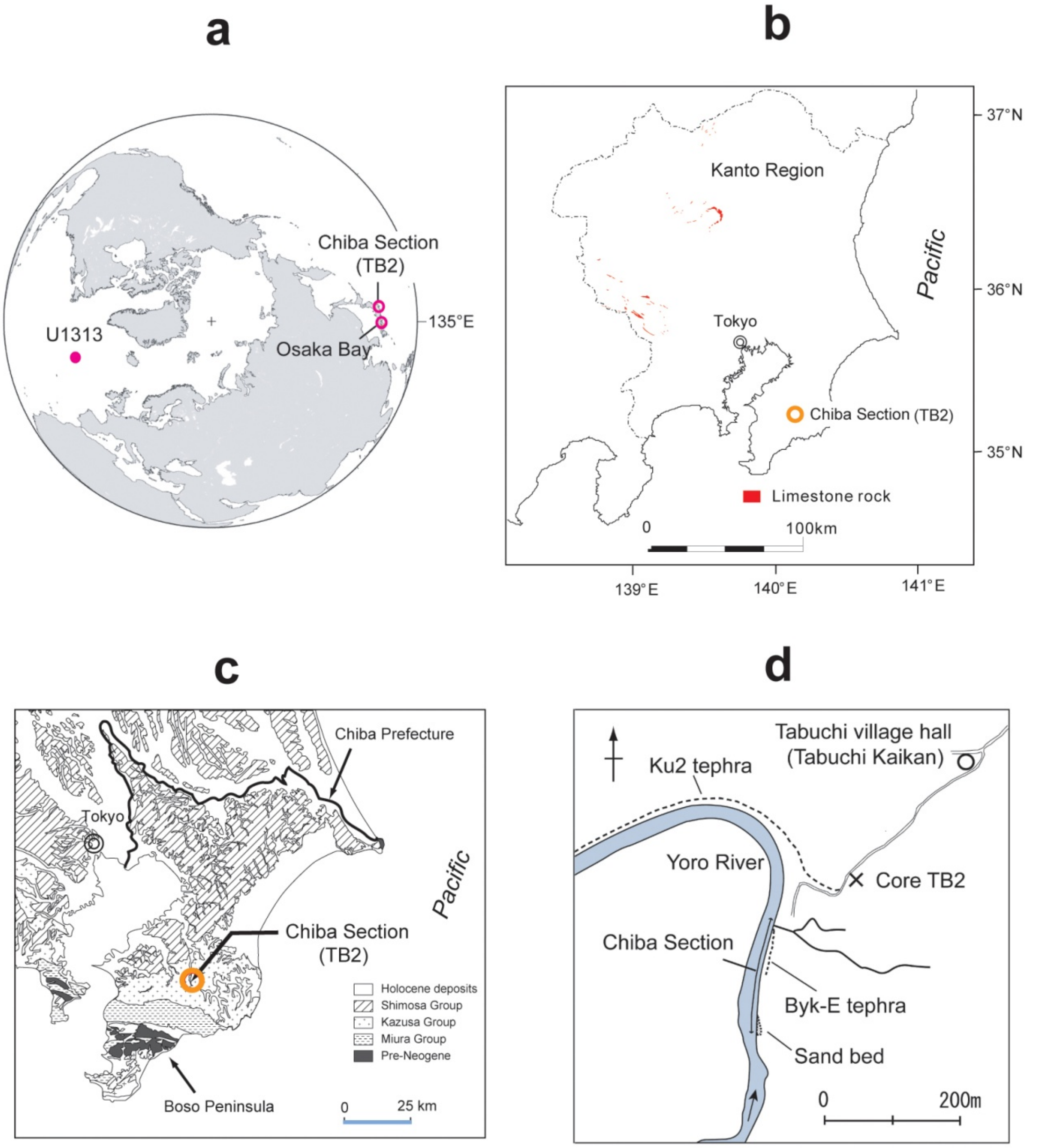

Figure S1. Maps of data localities. (a) Locations of the Chiba Section (core TB2), Osaka Bay and IODP site U1313. The map is drawn using the GMT version 4 (http://www.soest.hawaii.edu/gmt/). (b) Distribution of limestone rocks in the Kanto Region depicted from Seamless digital geological map of Japan[14]. (c) Geological map of the Chiba Section region (core TB2) [14]. (d) Map of the Chiba Section locality, a candidate for the GSSP of the Early-Middle Pleistocene boundary[15]. The cross shows the site of core TB2.

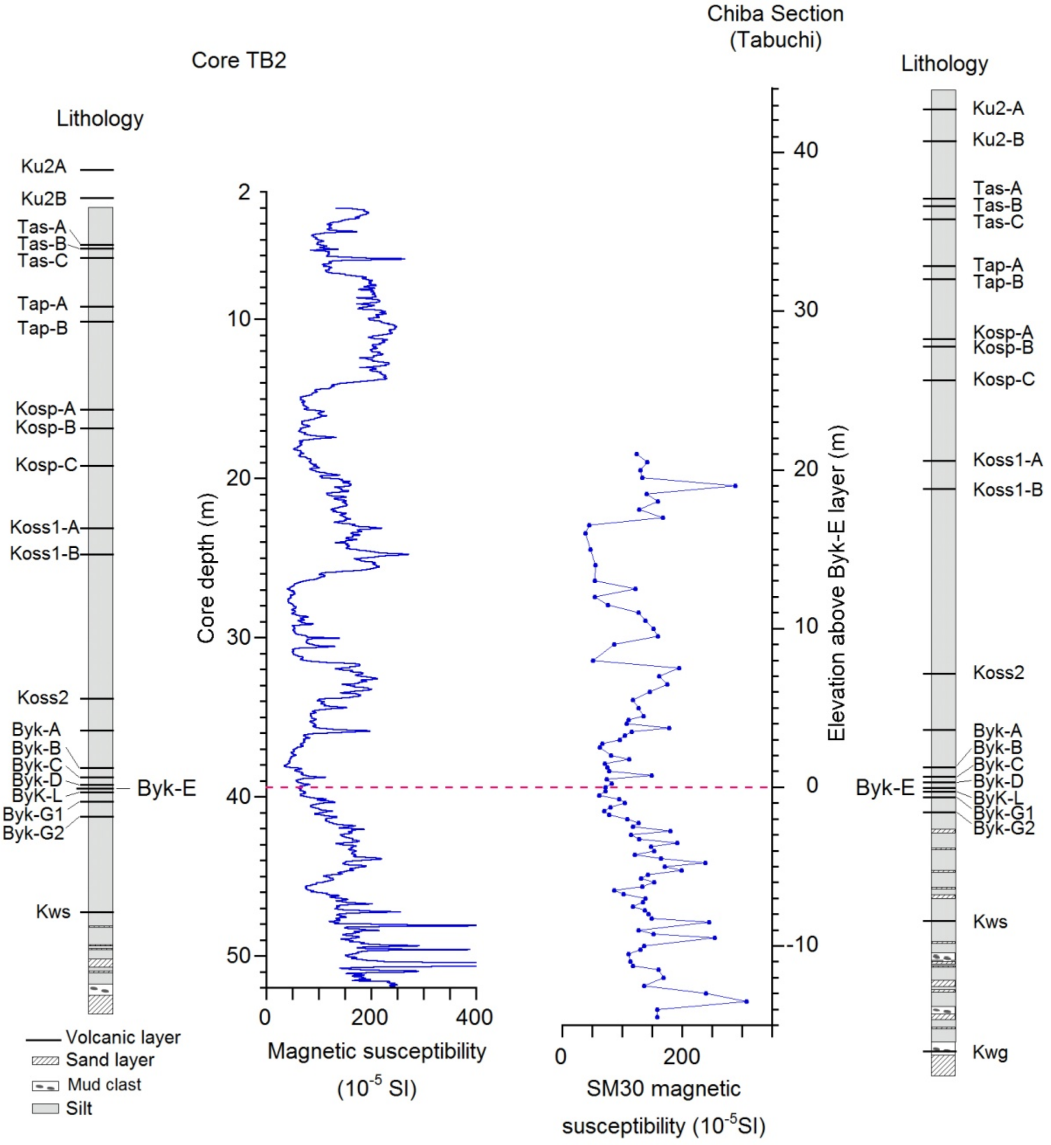

Figure S2. Lithostratigraphy and magnetic susceptibility of core TB2 and the Chiba Section. The magnetic susceptibility for core TB2 is after Ref. 6. The magnetic susceptibility for the Chiba Section was measured in the field using a SM30 ZH-Instrument portable magnetic susceptibility meter. The elevation range of magnetic susceptibility data agrees with that for the benthic oxygen isotope data in Supplementary Fig. S7. The names of tephra layers are after Ref. 16.

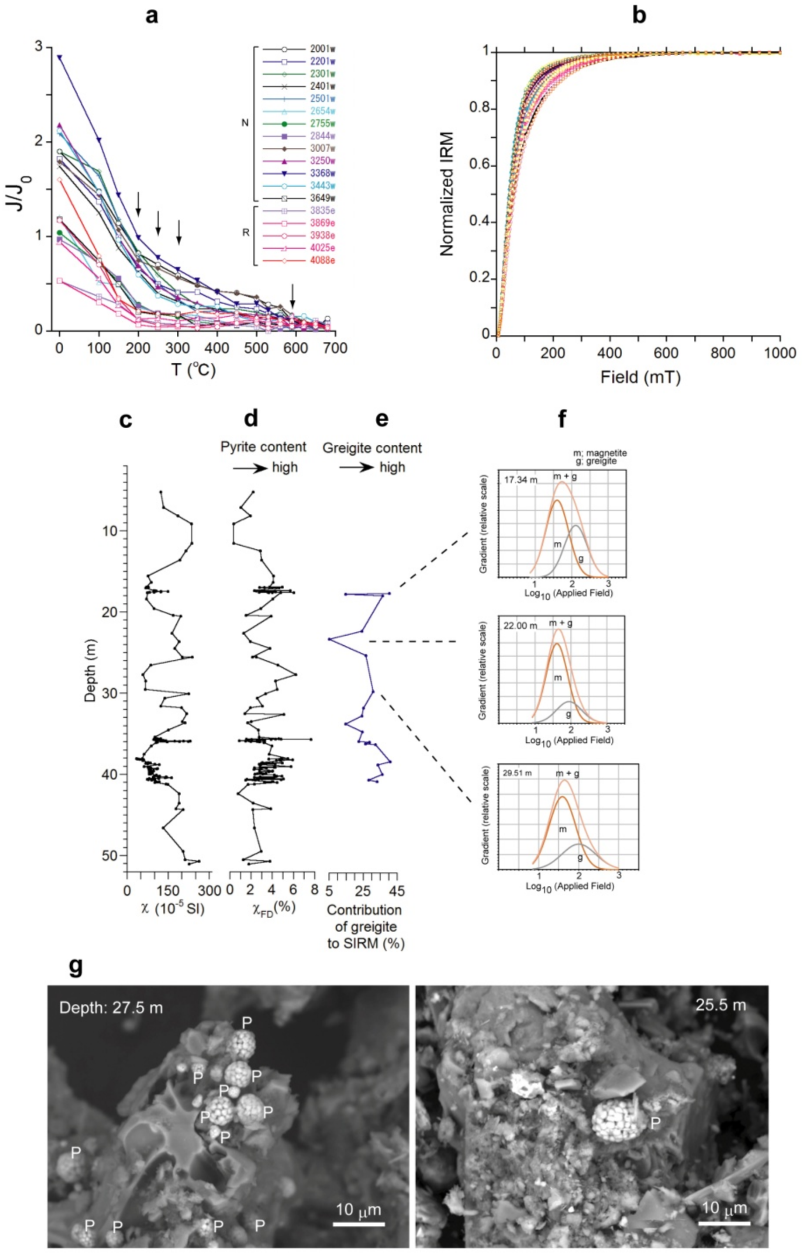

Figure S3. Magnetic results. (a) Natural remanent magnetization intensity decrease with temperature (after Ref. 6). N and R represent normal and reverse polarities, respectively. The arrows show unblocking temperatures. (b) Isothermal remanent intensity acquisition experiments. (c) Magnetic susceptibility (χ). (d) Frequency dependence of magnetic susceptibility (χ_{FD}). (e) Contribution of greigite to SIRM from the IRM component analysis. (f) Examples of the IRM component analysis. (g) SEM images of framboidal pyrite for low χ (left) (27.5 m in depth) and high χ (right) (25.5 m in depth) sediments.

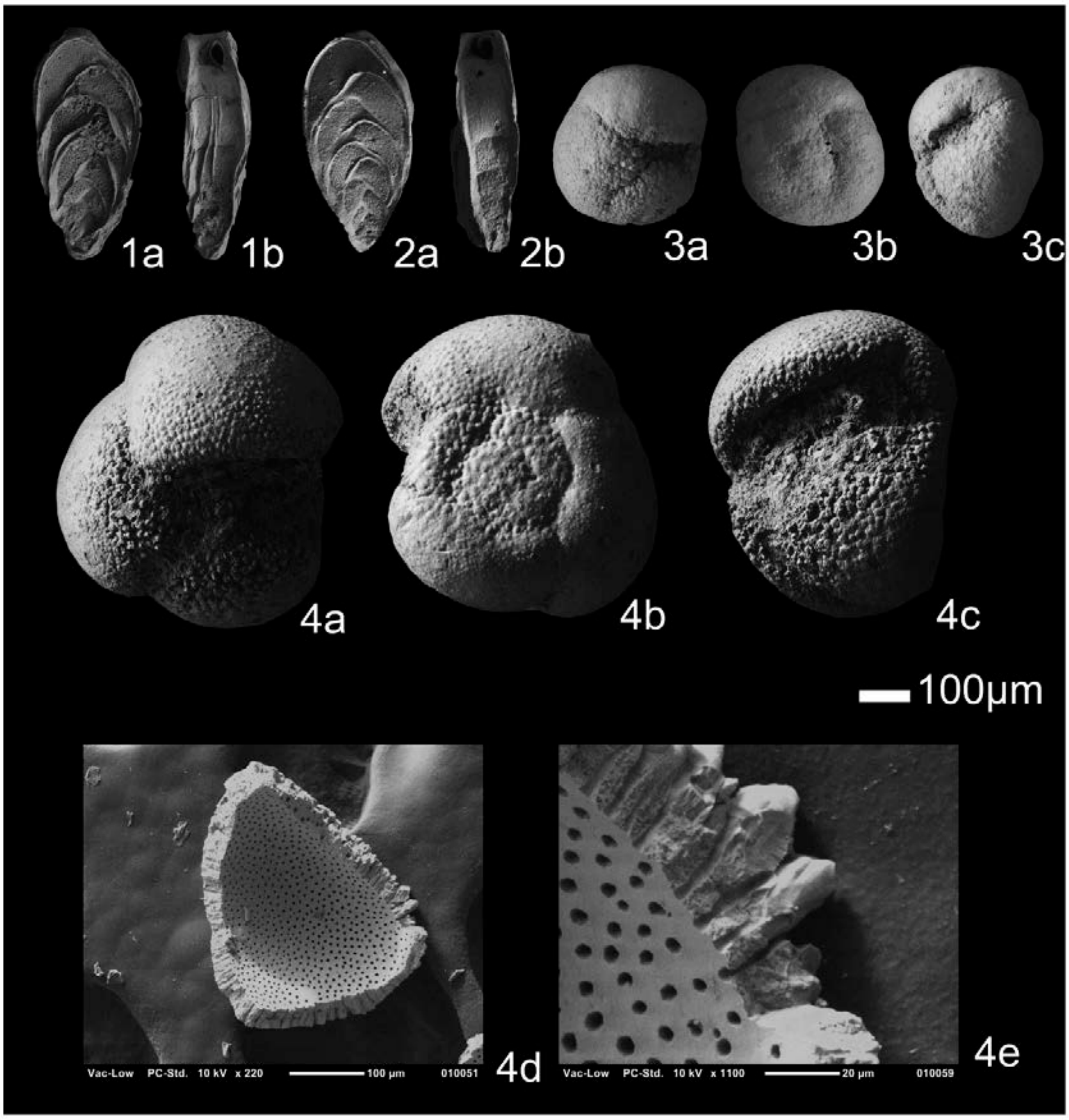

Figure S4. Scanning electron microphotographs of foraminiferal specimens. 1a, b and 2a, b: *Bolivinita quadrilatera*, sample TB11C. 3a–c: *Globorotalia inflata* (small adult individual), sample TB11C. 4a–e: *Globorotalia inflata* (large adult individual), sample TB31C. 4d and 4e show a processed fragment for isotope analysis.

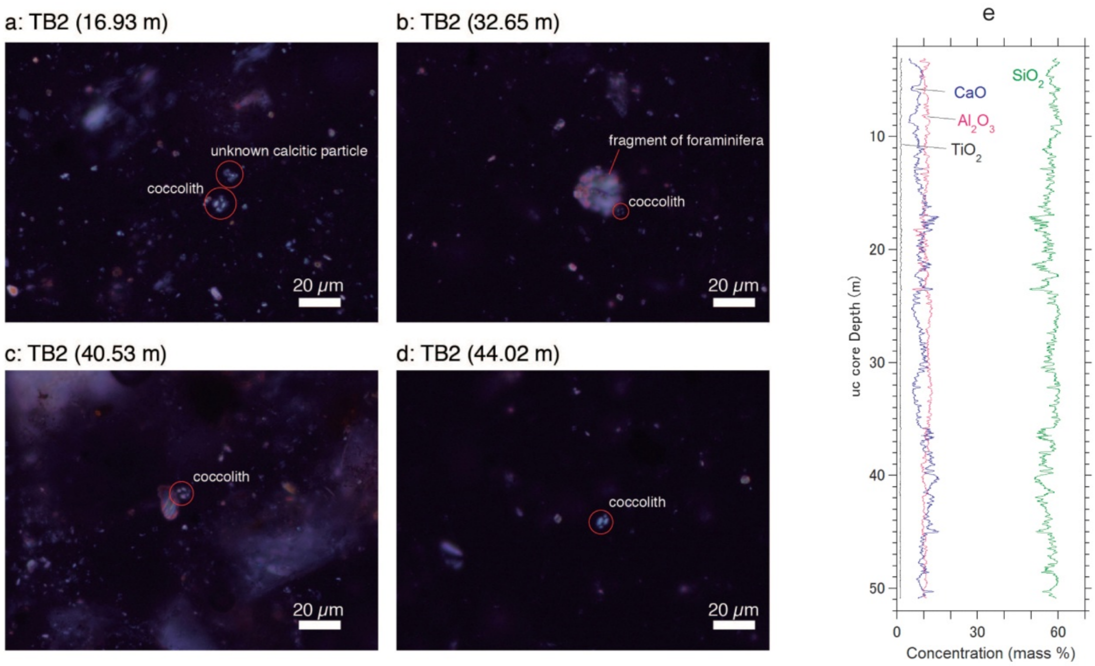

Figure S5. Optical photographs of bulk sediment samples (a-d) and sedimentary bulk major element concentrations (e). A polarizing microscope (Olympus CX31), 400 magnification, was used for taking photographs of the sediment samples from 16.93 m (a), 32.65 m (b), 40.53 m (c), and 44.02 m (d) depth. Red circles show calcite particles, which are dominantly coccolith and rarely foraminifera. Contents of coccolith estimated with color index are 11 % (a), 2 % (b), 10 % (c), and 6 % (d). The sedimentary bulk major element concentrations are calculated from the XRF data of the u-channel sample. 11 point moving average data are plotted for SiO_2 (green line), Al_2O_3 (red line), CaO (blue line), and TiO_2 (black line).

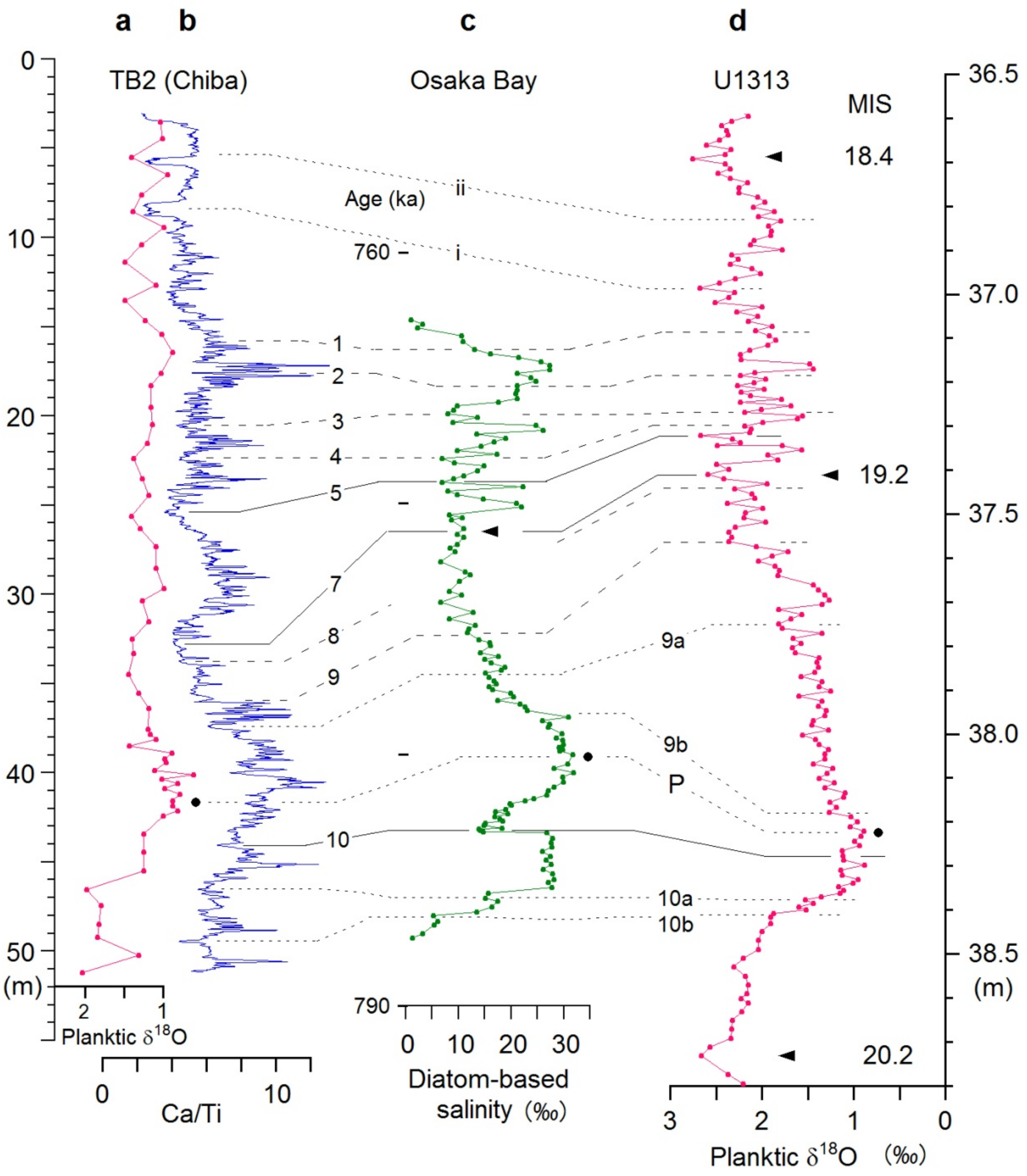

Figure S6. Comparison of paleoceanic environment records. (a), (b) Vertical plots of planktic $\delta^{18}O$ and Ca/Ti ratio of core TB2 from the Chiba Section. (c) Age plot of salinity estimated from diatom data from Osaka Bay, southwest Japan[17]. (d) Vertical plot of planktic $\delta^{18}O$ from IODP site U1313 in the mid-latitude North Atlantic[18]. The numbers 1 to 10 show the oxic events, and “P” the highest sea-level of highstand MIS 19.3. The tie-lines named ii, i, 9a, 9b, 10a, and 10b show sea-level lowstands and/or cooling events used for correlations. The tie-lines ii and i are determined based on an extension of the benthic $\delta^{18}O$ record from the Chiba Section covering the period until at least the MIS 18.4 lowstand[19].

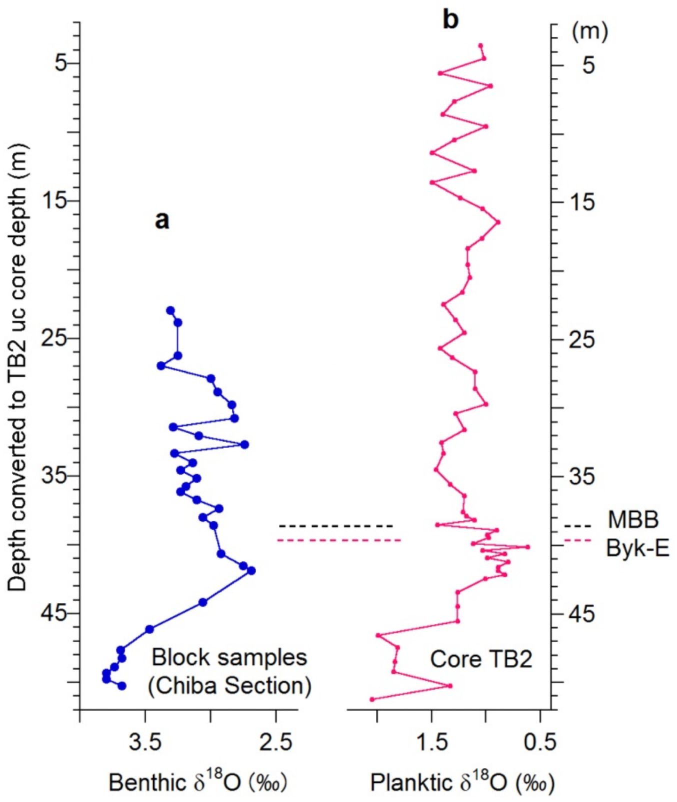

Figure S7. Vertical plots of the benthic $\delta^{18}O$ (a) and planktic $\delta^{18}O$ (b) data. The benthic data are from block samples collected at the Chiba Section. The depth scale of benthic $\delta^{18}O$ is converted to the u-channel core depth, based on the tephrostratigraphy and magnetic susceptibility data.

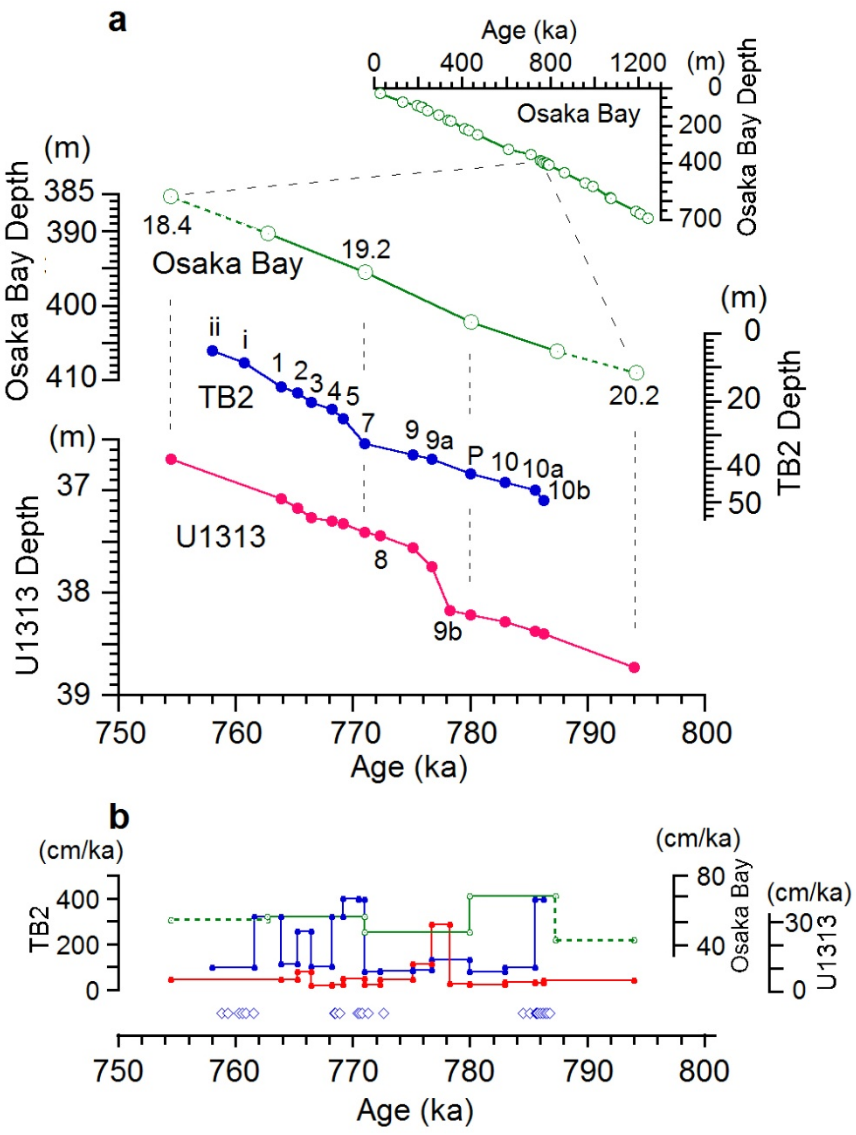

Figure S8. Age models for core TB2 and the U1313 data, based on the correlations in Fig. S6. (a) Depth versus age plots of age control points. The age control points for Osaka Bay are from Ref. 17, and those for core TB2 and U1313 are the oxic events, sea-level lowstands and/or cooling events (for details, see Method and Supplementary Fig. S6). (b) Mean accumulation rates between age control points, for the TB2 (blue line), Osaka Bay (green line), and U1313 (red line) data. The dotted line segments of the Osaka Bay plot are outside the MIS 19 marine interval. The open diamonds represent the horizons in core TB2 yielding the diatom species *Actinoctclus ingens*[9].

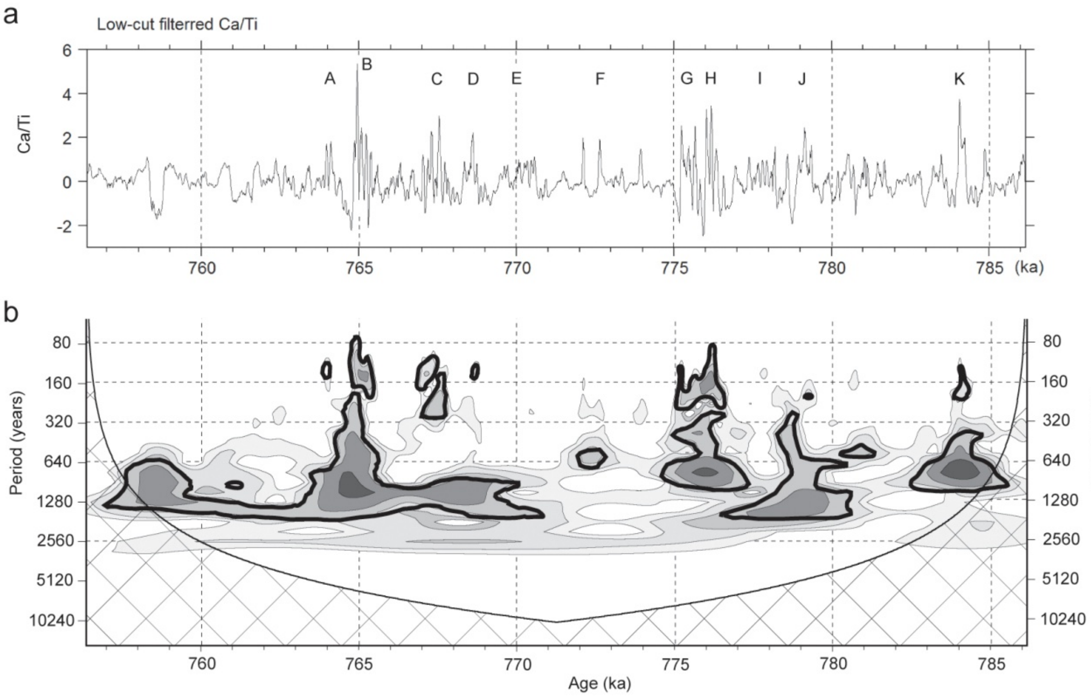

Figure S9. Wavelet power spectrum of the biogenic calcium carbonate content (Ca/Ti) from core TB2. (a) Low-cut filtered Ca/Ti data was used for wavelet analysis. 101 point (1010 yr) moving average values are subtracted from the original data (Fig. 2c) constructed with a 70-yr moving window. Consequently, the data are equivalent to those subjected to a band pass (70 to 1010 yr in periodicity) filter. (b) The local wavelet power spectrum of filtered data (a) using the Morlet wavelet. The left axis is the Fourier period (in yr) corresponding to the wavelet scale on the right axis. The bottom axis is age (ka). The shaded contours are at 2.5, 5, 10, 20, and 40. The thick contours enclose the regions of greater than 95 % confidence from a red-noise process with a lag-1 coefficient of 0.72[20]. The cross-hatched regions indicate the cone of influence, where zero padding reduces the variance.

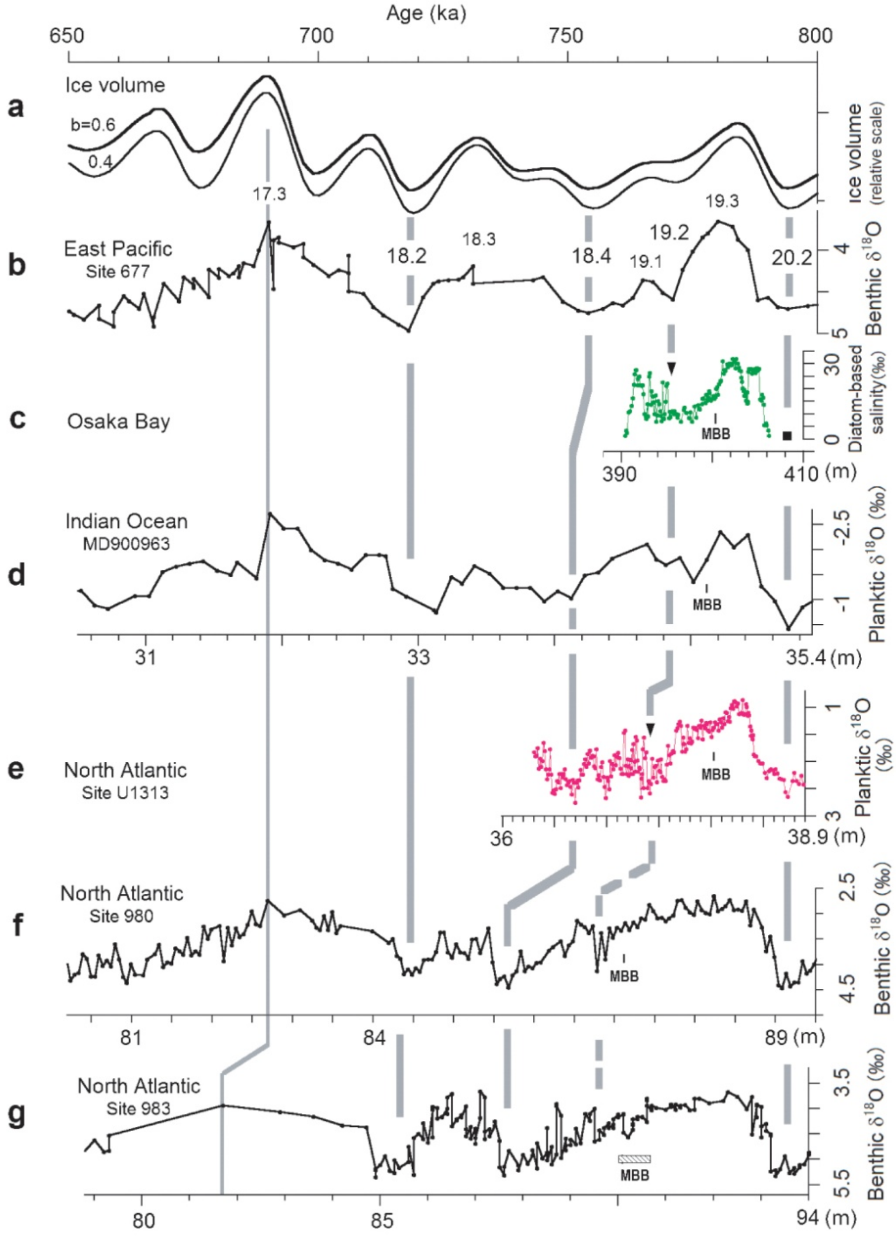

Figure S10. Comparison of marine oxygen isotope and sea-level proxy data. Age plots of (a) ice volume variations calculated with nonlinearity parameter b=0.6 and 0.4[11], and (b) oxygen isotope data from the East Pacific[21]. Depth plots of diatom sea-level proxy data from Osaka Bay[17] (c), and oxygen isotope data from (d) the Indian Ocean[22], (e) the mid-latitude North Atlantic[18], and (f), (g) two high-latitude sites in the North Atlantic[23,24]. Common features correlated with ice volume maxima (MIS 18.2, 18.4, 19.2, and 20.2) are tied by thick lines, and that with ice volume minima (MIS 17.3) by a thin line. MBB represents the Matuyama-Brunhes magnetic polarity boundary. The MBB in (g) shows a polarity transition zone.
